# Self-management of health dilemmas among Chinese distant-water fishing vessel crew: a descriptive qualitative study based on Orem’s self-care theory

**DOI:** 10.3389/fpubh.2026.1928192

**Published:** 2026-09-22

**Authors:** Dongdong Wu, Shilian Chen, Meiyuan Dong, Yin Lin, Boer Yan

**Affiliations:** 1 Department of Pain Medicine, Zhoushan Hospital, Wenzhou Medical University, Zhoushan, China; 2 Department of Neurology, Zhoushan Hospital, Wenzhou Medical University, Zhoushan, China; 3 Department of Nursing, Zhoushan Hospital, Wenzhou Medical University, Zhoushan, China; 4 Intensive Care Unit, Zhoushan Hospital, Wenzhou Medical University, Zhoushan, China

**Keywords:** crew, dilemmas, distant-water fishing, health self-management, Orem’s self-care theory, qualitative research

## Abstract

**Background:**

Distant-water fishing is one of the world’s most hazardous occupations, characterized by extreme isolation from medical facilities, prolonged voyages, and high-intensity labor. Despite documented risks, qualitative evidence on how Chinese crew members manage their health during extended voyages remains scarce.

**Objective:**

This study explored health self-management dilemmas among Chinese distant-water fishing vessel crew members, guided by Orem’s Self-Care Theory.

**Methods:**

A descriptive qualitative design was employed. Purposive and maximum variation sampling recruited 19 crew members from squid-jigging vessels in Zhoushan, China (February–June 2026). Face-to-face semi-structured interviews were conducted in private spaces. Data were analyzed using thematic analysis with NVivo 15.0.

**Results:**

Three themes emerged: (1) inadequate distant-water medical resources (lack of professional care, insufficient supplies, transport delays, hierarchical pressure blocking help-seeking); (2) high-intensity operations (disrupted work-rest cycles, chronic exhaustion, monotonous isolated life with emotional distress, willingness to manage health yet constrained by conditions); and (3) weak interpersonal support (utilitarian peer relationships, formalized family support with “reporting good news while concealing bad news”). Structural conditions shaped crew members’ health management practices and limited available options.

**Conclusion:**

Health self-management dilemmas are structurally rooted, produced by inadequate medical resources, hazardous working conditions, and functionally decomposed support systems. Recommendations include independent medical reporting channels, enforced work-rest protections, accessible medication guidance, enhanced first-aid capacity, and telemedical services via satellite communication.

## 1 Introduction

Distant-water fishing is one of the world’s oldest and most hazardous occupations (1, 2). Unlike merchant shipping, distant-water fishing vessels operate in geographically isolated areas—primarily the Pacific, Indian, and Atlantic Oceans, as well as Antarctic waters—often remaining at sea for 6–24 months per voyage (3). By the end of 2024, China had approximately 2,551 approved distant-water fishing vessels, of which 1,498 were operating on the high seas, producing an annual catch of around 2.33 million tonnes (4). Zhoushan, China’s largest distant-water fishing production base, alone accounted for nearly 700 vessels and over 2,300 practitioners by 2024 (5, 6). These vessels are characterized by extreme occupational hazards: prolonged physical isolation from land-based medical facilities, limited communication infrastructure, high-intensity labor driven by catch-dependent wages, and exposure to severe weather and heavy machinery operation. Civil society organizations such as Human Rights at Sea (HRAS) have documented individual cases of crew members and fisheries observers being denied medical care, subjected to violence, and deprived of independent communication channels on distant-water fishing vessels, including incidents where fishers were refused appropriate shore-based medical treatment or where medical assistance was withheld due to concerns about costs or operational disruption (7). While such case reports demonstrate the existence and types of potential gaps in human rights protections in the sector, they should not be interpreted as incidence statistics. Recent regional surveys indicate that forced labor risks persist in Southeast Asian fisheries, with approximately 13% of migrant workers facing forced labor conditions, rising to 20% among fishers on distant-water tuna vessels where oversight and enforcement are limited (8). While such case reports demonstrate the existence and types of potential gaps in human rights protections in the sector, they should not be interpreted as incidence statistics; quantitative data on the frequency and representativeness of these abuses remain limited and require supplementation with official statistics from the ILO, IMO, FAO, or peer-reviewed epidemiological studies.

The occupational fatality rate in commercial fishing is exceptionally high. Globally, fishing-related deaths exceed 100,000 annually, with fatality rates in some regions reaching approximately 80 per 100,000 fishers—far surpassing those of land-based occupations (9). In the United States, fishing and hunting workers had a fatal injury rate of 88.8 per 100,000 full-time equivalent workers in 2024, approximately 27 times the national average of 3.3 per 100,000 across all industries (10). Traumatic injuries, musculoskeletal disorders from repetitive heavy labor, and cardiovascular diseases linked to irregular work-rest cycles and high-salt diets are prevalent among fishing crews (11–13). Psychologically, prolonged social isolation, monotonous living conditions, and high production pressure render these crews vulnerable to occupational stress, loneliness, anxiety, and depression (14). Surveillance data from England and Wales further confirm that distant-water fishing exhibits the poorest overall health status and the highest prevalence of long-term work-limiting health conditions among all industries (15).

International labor standards have attempted to address these systemic risks. The International Labor Organization (ILO) Work in Fishing Convention, 2007 (No. 188), which entered into force on November 16, 2017, establishes binding minimum standards for ratifying States regarding medical care at sea and ashore, occupational safety and health, rest periods, and living conditions (16). Articles 29 requires ratifying States to ensure that fishing vessels carry appropriate medical equipment and supplies, have at least one crew member trained in first aid, maintain radio or satellite communication for medical advice, ensure fishers’ right to timely medical treatment and transfer ashore for treatment in cases of serious injury or illness; Article 21 provides for repatriation; Articles 31–33 establish occupational safety and health protection measures (16). Despite these binding international minimum standards, significant implementation gaps persist in the distant-water fishing sector. As of 2026, C188 has been ratified by only 27 countries, reflecting a broader challenge in the global governance of fishing labor standards. China, the world’s largest distant-water fishing nation, has not yet ratified this Convention (17). China has nevertheless established domestic regulations governing distant-water fishing operations. The Measures for the Administration of Fishery Crew (2014) impose binding duties on owners and operators of Chinese-flagged fishing vessels: Article 6 mandates basic safety training including first aid; Articles 32–34 require owners to provide medical supplies, maintain crew health records, ensure timely treatment of injury or illness, and guarantee sufficient rest periods; Articles 35–39 establish administrative supervision and public reporting channels; and Article 47 provides penalties for failure to treat sick or injured crew promptly (18). These domestic provisions do not establish protections equivalent to C188, nor do they guarantee effective enforcement; however, they demonstrate that regulatory duties exist on paper, and the gap lies primarily in compliance and enforcement rather than in a complete absence of rules. The absence of ratification means these domestic protections are not subject to the same international oversight and enforcement mechanisms as those established under the MLC for merchant vessels, and does not indicate a lack of domestic regulatory attention to crew welfare.

Onboard medical facilities on fishing vessels frequently lack critical equipment such as electrocardiographs and defibrillators, and diagnosis often relies solely on symptom description (19). A 2024–2025 study further confirmed that seafarers on cargo ships typically had minimal medical provisions, with healthcare responsibility falling to officers with limited medical training, leading to underreporting of symptoms and self-medication (20). The International Medical Guide for Ships, though widely distributed, is considered cumbersome and difficult to navigate during emergencies (21). In 2023, the International Chamber of Shipping published a new practical medical guide—the International Medical Guide for Seafarers and Fishers—specifically designed for non-medical professionals on board ships and fishing vessels, featuring 3D visual aids, action cards, and an updated medicine chest (22). However, its adoption on Chinese distant-water fishing vessels remains limited. For these vessels specifically, medical evacuation delays are critical. When serious illness occurs, transport back to China can take nearly a month, and production tasks often take priority over crew members’ health needs. Limited access to healthcare itself constitutes an occupational risk factor—seafarers face a 42% higher risk of hospitalization for community-acquired pneumonia (IRR = 1.42, 95% CI: 1.15–1.77) (23), underscoring how inaccessibility of healthcare directly compromises health outcomes in maritime occupations.

Despite these documented risks, empirical evidence specifically examining the health self-management experiences of Chinese distant-water fishing vessel crew members remains scarce. Existing research on crew members’ health has predominantly employed quantitative cross-sectional designs, focusing on disease incidence and injury rates (11, 12, 15), thereby describing “what diseases crew members have” from an external perspective. Few studies have explored how crew members manage their health in real ocean-going contexts or the practical difficulties they encounter. International regulatory frameworks have increasingly recognized mental health as a safety and governance issue rather than merely an individual adaptation challenge. The IMO Maritime Safety Committee, at its 108th session in May 2024, adopted amendments to the STCW Code (Resolution MSC. 560 (108)) introducing mandatory training for all seafarers on preventing and responding to violence and harassment, including sexual harassment, bullying and sexual assault, which entered into force on 1 January 2026. Concurrently, the ILO-IMO Joint Tripartite Working Group has addressed violence, harassment, and sexual assault at sea, requiring shipowners to provide timely medical and mental health care to victims (24). However, these frameworks primarily target merchant shipping. They cannot be assumed to be equally applicable to distant-water fishing vessels, which face distinct structural vulnerabilities, including catch-dependent remuneration systems, concentrated authority held by captains and vessel owners, limited communication infrastructure, ambiguous employment relationships, and overlapping jurisdictions that may create even larger gaps in medical and psychological support. A recent scoping review that systematically mapped research on fishermen’s mental health identified a severe shortage of high-quality empirical studies, with designs dominated by cross-sectional surveys and an absence of validated intervention programs; the authors explicitly called for qualitative research to understand the lived experiences underlying health vulnerabilities (25). In the Chinese context, where the distant-water fishing fleet is among the world’s largest, this evidence gap is particularly acute. No qualitative studies have specifically investigated how Chinese distant-water fishing crews navigate health dilemmas related to medical security, occupational environments, and social support during extended voyages.

To understand the underlying mechanisms of crew members’ self-management of health dilemmas, this study adopts Orem’s Self-Care Theory as an analytical framework. The theory comprises four core constructs. Therapeutic self-care demands encompass the totality of self-care actions required to maintain health and wellbeing, including managing injuries and illnesses, maintaining chronic disease management, and preserving mental health. Self-care agency is an individual’s capability to engage in self-care, shaped by basic conditioning factors—internal and external determinants such as access to the health care system, environmental conditions, and resource adequacy. When self-care agency is insufficient to meet therapeutic self-care demands, a self-care deficit arises, indicating the need for nursing intervention (26). Orem’s supportive-educative nursing system refers to the professional assistance provided to persons capable of performing or learning to perform self-care, with the nurse’s role being to support and educate the individual toward autonomous self-care (26). In the distant-water context, crew members face objective therapeutic self-care demands; however, their self-care agency is continuously undermined by profoundly compromised basic conditioning factors: the health care system is virtually absent, the occupational environment is chronically hazardous, and essential resources—including medical supplies, communication infrastructure, and rest facilities—are inadequate. Notably, in the absence of professional nursing support, peer relationships and family communication operate as basic conditioning factors that shape self-care agency, rather than as components of a nursing system in Orem’s original formulation: their functional compromise erodes self-care agency and contributes to self-care deficit. This “demand-agency” imbalance constitutes the crew’s self-care deficit state. Consequently, the self-care deficit observed among crew members cannot be understood merely as individual failure or insufficient self-care motivation; rather, it reflects systemic structural barriers that actively undermine self-care agency (24). Previous studies have applied Orem’s Self-Care Theory to explore the self-care needs of various occupational populations, confirming its applicability in understanding occupation-related self-care dilemmas. However, its application in the specific occupational context of crew members on distant-water fishing vessels remains underexplored.

This study uses Zhoushan as the survey site—the city hosts China’s largest distant-water fishing production base, accounting for more than one-third of the national annual catch (5)—and focuses on the health management dilemmas faced by crew members aboard Chinese distant-water fishing vessels. Adopting a descriptive qualitative research approach centered on three core dimensions of Orem’s Self-Care Theory framework, this study asks: What are the therapeutic self-care demands in the distant-water environment? What factors undermine crew members’ health self-management capacity? What supportive-educative nursing systems are available, and how do their inadequacies contribute to self-care deficits? What self-care deficit experiences do crew members have? By answering these questions, this study aims to explore the multidimensional dilemmas they face in health self-management and to provide empirical evidence for telehealth services, onboard health education, and psychological support programs.

## 2 Methods

### 2.1 Study design

This study employed a two-layer analytical framework. The first layer is a normative and institutional analysis examining the regulatory protections afforded to distant-water fishing vessel crew members under international labor standards (primarily ILO C188), domestic regulations, and vessel owner obligations. The second layer applies Orem’s Self-Care Theory to analyze crew members’ lived experiences of health self-management. The two layers are integrated through Orem’s concept of “basic conditioning factors,” which explicitly recognizes that self-care agency is shaped by healthcare system factors, environmental conditions, and resource adequacy—factors that are themselves determined by regulatory and institutional arrangements (26). Table 1 presents the integrated theoretical-regulatory framework guiding this study. It is a theory-driven qualitative research approach with a qualitative descriptive design to explore the self-management of health dilemmas of crew members on distant-water fishing vessels. A qualitative descriptive design is suitable for directly exploring individuals’ experiences, perceptions, and meanings regarding a specific phenomenon (27). This design is appropriate for presenting participants’ authentic voices in natural settings, particularly for exploring health issues that have not been fully investigated. Two nursing qualitative research experts provided methodological guidance. To ensure comprehensive reporting, this study adhered to the Consolidated Criteria for Reporting Qualitative Research (COREQ) checklist (28). The full checklist is available in Supplementary material S2.

**Table 1:** Theoretical-regulatory framework.

Orem’s self-care concept	Regulatory/institutional dimension	Relevant ILO C188 provisions and other standards
Therapeutic self-care demands	Healthcare system accessibility; medical examination standards	Art. 10–12: Medical examination of fishers; Art. 29: Medical equipment and supplies, radio/satellite medical advice, right to treatment ashore; Art. 30: Additional medical requirements for vessels ≥24 m
Self-care agency (environmental conditioning factors)	Occupational safety and rest regulation; port-State control	Art. 13–14: Manning and minimum rest periods; Art. 31–33: Occupational safety and health protection; Art. 43: Port State control
Self-care agency (resource adequacy)	Onboard medical resource allocation; vessel owner/employer responsibilities	Art. 8: Responsibilities of fishing vessel owners and skippers; Art. 29(a)-(d): Medical equipment, medicines, trained crew, radio/satellite communication
Supportive-educative nursing system*	Social protection and grievance mechanisms; inspection and enforcement	Art. 16–20: Fisher’s work agreements; Art. 21: Repatriation; Art. 23–24: Payment of fishers; Art. 40: Compliance and enforcement (including complaint procedures); Art. 41–42: Inspection mechanisms
Self-care deficit	Flag State ratification and enforcement gap; ILO recommendations and domestic legal obligations	Art. 1–4: Scope and definitions; Art. 6: Implementation; Art. 22: Flag State responsibilities; Art. 39: Health protection and medical care (owner liability); Art. 43: Port State control; ILO R199 (2007): Implementation guidance; domestic regulations

Orem’s core concepts (26) are mapped to corresponding regulatory dimensions and ILO C188 provisions. Vessel owner responsibilities (Art. 8), flag-State supervisory obligations (Art. 6, 22, 40–42), port-State control mechanisms (Art. 43), and medical examination standards (Art. 10–12) are explicitly incorporated to ensure comprehensive normative coverage.

### 2.2 Participants and setting

Purposive sampling was conducted in Zhoushan City, Zhejiang Province, from February to June 2026. Zhoushan is China’s leading city for distant-water fishing and hosts the country’s largest distant-water fishing production base. By the end of 2024, Zhoushan had 39 distant-water fishing enterprises, 693 distant-water fishing vessels, and approximately 16,000 distant-water fishing vessel crew members (29). This large, diverse crew population provided an ideal sampling frame for this study. The researchers contacted two distant-water fishing enterprises in Zhoushan through a district-level pelagic fishery association. The Association and enterprises served solely as contact facilitators; they did not participate in participant selection, were not informed of which crew members participated, and did not receive interview transcripts or findings. The research team directly approached potential participants on-site at shore-side facilities within 3 days prior to departure or within 3 days after return to shore. One eligible participant declined to participate due to time constraints; this declination was not communicated to the Association or enterprises, and did not require the provision of reasons. A combination of purposive and maximum variation sampling was used to recruit 19 crew members from distant-water fishing vessels with recent long-distance ocean-going experience.

Maximum variation sampling was also employed to ensure that the sample comprehensively reflected the health self-management experiences of crew members with different characteristics. The core logic of maximum variation sampling is that if common themes can be identified among highly diverse individuals, the themes have greater explanatory power (29). Based on a literature review and preliminary understanding of the crew population, the research team identified the following variation dimensions: (1) years of experience in distant-water fishing (<5 years, 5–9 years, ≥10 years), to cover different career stages from novice to veteran; (2) job rank (management-level crew vs. non-management-level crew), to reflect differences in onboard power structure and health decision-making capacity; and (3) age (<35 years, 35–49 years, ≥50 years), to encompass health needs and coping strategies across different life stages. During recruitment, priority was given to individuals who could fill gaps in existing sample characteristics.

To minimize the risk of coercion, interviewers explicitly stated—both in the written informed consent and orally at the start of each interview—that participation was voluntary, would not affect employment, contracts, or future voyage assignments, and that participants could withdraw at any time without consequences. Each participant received a 200 RMB stipend for their time, provided by the research team upon completion of the interview; this was disclosed in the informed consent process. An audio recording was used with explicit consent; participants could pause or stop recording at any time.

Inclusion criteria were: (1) currently serving as a distant-water fishing vessel crew member; (2) aged ≥18 years; (3) able to communicate effectively in Mandarin; and (4) willing to participate and provide written informed consent. Exclusion criteria were: (1) presence of severe cognitive impairment or mental illness that would prevent understanding of interview content or provision of valid information; and (2) hospitalization for acute illness or injury that would preclude completion of the interview.

Sample size was determined based on the principle of information power (30), which suggests that samples can be smaller when the study aim is narrow, participants hold relevant experiences, and a strong theoretical framework is applied. Data saturation was operationally defined as the point at which three consecutive interviews yielded no new, substantively relevant codes or themes. The research team conducted interim saturation assessments after interviews 5, 10 and 15. After interview 10, new codes were still emerging. After interview 15, no new themes had emerged in three consecutive interviews (interviews 13–15). Four additional interviews (16–19) were conducted to confirm saturation, yielding no substantively new codes. The research team judged that additional data would not further deepen or diversify the existing themes.

During the recruitment period, one eligible participant declined to participate due to time constraints, and no participants withdrew after providing written informed consent.

To minimize social desirability bias and fear of retaliation, the research team employed several safeguards. First, management-level and non-management-level crew members were recruited through the same contact channels but approached separately during on-site visits to avoid hierarchical influence on participation decisions. Second, the research team explicitly communicated to all participants—both in written informed consent and orally—that the Association and enterprises would not know which individuals participated, that participation would not affect employment or future voyage assignments, and that withdrawal would carry no consequences. Third, interviews were conducted in private spaces away from company premises with no company representatives present. Despite these measures, we acknowledge that participants may still have exercised self-censorship when disclosing sensitive information, such as denied medical care or hierarchical pressure, particularly given the concentrated authority structure on board. We addressed this by using open-ended questions, allowing participants to control the depth of disclosure, and following a standardized ethical protocol when sensitive information emerged.

### 2.3 Interview guide development

A semi-structured interview guide was developed based on Orem’s Self-Care Theory (26), a literature review, and the research team’s expertise. The interviews were structured around the three core concepts of Orem‘s theory, with specific questions presented in Table 2.

**Table 2:** Theoretical dimensions of the interview guide and corresponding questions.

Theoretical dimension	Interview questions
Therapeutic self-care demands (perceived health threats and self-care needs)	1. What health problems do you worry about most during distant-water operations?
	2. In what situations do you think you are most likely to get injured or become ill on board?
	3. What do you think you need to do to maintain your health while on board?
Self-care agency (environmental and occupational factors constraining self-care capacity)	4. How is your work-rest schedule arranged on board? How does this rhythm affect your physical health or mood?
	5. What do you know about the medical kit or first-aid equipment on board? Do you use them in daily practice?
	6. If you or a colleague feels unwell on board, how do you usually handle it? Who do you turn to for help?
Self-care deficit (experiences when self-care needs cannot be met)	7. Have you encountered or heard of any situations on board where illness or injury was particularly difficult to manage? What happened?
	8. When you feel emotionally distressed on board, how do you usually cope? Is there anyone you can talk to?
	9. In your opinion, what aspects of the current onboard conditions are insufficient for protecting your health?

All questions were open-ended, and the researchers asked further probing questions based on participants’ responses. The interview guide was finalized after discussions within the research team and pilot testing, and was flexibly adjusted during the formal interviews. The full interview guide is available in Supplementary material S1.

Before the formal interviews, WDD and CSL conducted pilot interviews with two crew members from distant-water fishing vessels who met the inclusion and exclusion criteria (non-formal study participants) to assess the relevance, clarity, and flow of the questions. The pilot interviews were conducted in a quiet, private space arranged by the research team in a venue unaffiliated with the participating enterprises, each lasting approximately 30–60 min. After the research team reviewed and discussed the audio recordings of the pilot interviews, minor adjustments were made to the wording and question sequence to enhance clarity and participant comfort, without substantively altering the core question content. The revised guide was finalized and consistently used in all subsequent formal interviews.

### 2.4 Data collection

Interviews were conducted either within 3 days prior to departure (*n* = 10) or within 3 days of participants’ return to shore (*n* = 9). Both groups provided retrospective accounts of their most recently completed voyage: return-to-shore interviews captured experiences while memory was fresh; pre-departure interviews captured experiences from the voyage just concluded, as participants had not yet embarked on a new voyage. Because both groups reported similar health management dilemmas—return-to-shore participants recalling past voyages and pre-departure participants anticipating upcoming ones—the two types of accounts were combined in the analysis without stratification by timing. All interviews were completed before crew members dispersed to their home provinces following completion of entry procedures. All interviews were scheduled during afternoon hours (13:00–17:00) to ensure participants were physically rested and mentally alert. They were conducted in private spaces arranged by the researchers, not in company offices or vessels. Locations included quiet rooms in shore-side facilities unaffiliated with the enterprises. No company representatives, supervisors, or management personnel were present during interviews. Face-to-face semi-structured interviews were used to collect data. All interviews were conducted in Mandarin Chinese (the participants’ native language). The interview guide was originally developed in Chinese. All interviews were conducted in Mandarin Chinese, audio-recorded, and transcribed verbatim in Chinese by WDD and CSL. Coding was performed in Chinese by WDD on all 19 transcripts, with CSL independently coding five transcripts (approximately 25%) in Chinese. Selected quotations were translated into English by WDD and back-translated into Chinese by CSL to check accuracy; discrepancies were resolved through discussion. The final translated quotations were reviewed by a bilingual methodology expert to ensure semantic equivalence. The English translation of the interview guide is available in Supplementary material S1. Before the interviews began, participants completed a paper-based demographic questionnaire collecting the following information: (1) age, gender, marital status, and education level; (2) occupation-related information (job title and years of experience in distant-water fishing). These data were used to describe sample characteristics and to provide a background reference for understanding individual differences in data analysis.

Prior to the formal interviews, the researchers explained the study’s purpose, the interviewer’s background, procedures, confidentiality measures, and principles of voluntary participation to each participant in detail. All interviews commenced after informed consent was obtained and were audio-recorded in their entirety. Participants were informed that they had the right to pause or stop the recording at any time. During the interviews, interviewers used open-ended questions to encourage participants to freely express their authentic experiences, dilemmas, and views regarding health self-management. Interviewers noted participants’ emotional tone and non-verbal cues to inform rapport and interview sensitivity; these observations were not systematically analyzed as primary data. All interviews were conducted in private spaces either before the participants’ departure or after their return to shore, with no one else present. Each interview lasted 30–60 min, with an average duration of approximately 35 min.

All interviews were conducted separately by WDD and CSL, and the two interviewers did not interview the same participant. WDD conducted 10 interviews and CSL conducted 9. We did not formally test for interviewer effects due to the small sample and because no participant was interviewed by both researchers; however, reflexive discussions after each interview and joint calibration of coding discrepancies were used to promote consistency. Interviewers had no prior personal or professional relationship with participants or enterprises. When participants disclosed sensitive information (e.g., denied medical care, delayed evacuation, hierarchical pressure), interviewers followed a standardized protocol: (1) pause to assess participant distress; (2) remind participants of confidentiality and their right to withdraw; (3) avoid probing questions that could retraumatize; (4) offer referral to hospital psychological counseling services; (5) document disclosures in research memos without identifying information. Both interviewers are female nursing researchers from island regions. WDD holds a master’s degree in nursing, and CSL holds a bachelor’s degree in nursing. Both have received training in qualitative research methods and have over 10 years of clinical nursing experience. The researchers had no personal or professional relationships with potential participants prior to the study.

After each interview, the research team engaged in reflexive discussions covering: (1) review and reflection on the interview process; (2) documentation of preliminary insights and potential themes emerging from the interview; (3) discussion of challenges encountered and coping strategies; and (4) identification of the researchers’ own presuppositions and their potential influence on the interviews. The content of these reflexive discussions was recorded in research memos for reference during subsequent data analysis.

### 2.5 Data analysis

All interviews were transcribed verbatim. The recordings were initially transcribed using the Get Note online transcription function, then manually checked and corrected sentence by sentence by the researchers to ensure consistency between the audio and the text. Another researcher independently reviewed the transcripts, and any discrepancies were resolved through joint listening to the recordings. Finally, the transcripts were returned to all 19 participants for confirmation of accuracy. This transcript confirmation was distinct from the subsequent member-checking of themes, which involved only three participants. The confirmed transcripts were imported into NVivo 15.0 (QSR International Pty Ltd., Burlington, MA, United States) for data management and coding.

Thematic analysis was used to organize, code, and categorize the data (31). The analysis process comprised six phases: (1) familiarization with the data; (2) generating initial codes; (3) searching for themes; (4) reviewing themes; (5) defining and naming themes; and (6) producing the report. WDD repeatedly read all transcripts to become familiar with the data and to gain an overall understanding, and then systematically coded all 19 transcripts based on this understanding. To ensure coding rigor, CSL independently coded five of the transcripts (approximately 25%). Coding concordance and discrepancies were calibrated and resolved through discussions within the research team, including a methodology expert. WDD then uniformly applied the final coding framework to the entire dataset. Codes with similar meanings were grouped to form initial sub-themes. The sub-themes were then reviewed for similarities and differences and further refined to ensure coherence and distinctiveness. The sub-themes were subsequently synthesized into final themes to answer the research questions. Throughout the theme development process, the research team maintained ongoing dialog with Orem’s Self-Care Theory. Themes directly corresponding to Orem’s core concept were labeled “theory-consistent themes.”

In contrast, themes that emerged from the data but were not explicitly covered by the theory were labeled as “data-emergent themes.” NVivo 15.0 was used to manage all codes, memos, and thematic structures. The research team maintained a complete audit trail of the coding process to ensure transparency and traceability of the analysis, as detailed in Supplementary material S3.

### 2.6 Trustworthiness and rigor

Multiple strategies were employed to ensure the credibility, transferability, and dependability of the study findings. In addition to the transcript confirmation conducted with all 19 participants, preliminary themes and findings were sent via telephone or WeChat to three participants (one from management-level crew and two from non-management-level crew, with years of experience in distant-water fishing covering <5 years, 5–9 years, and ≥10 years) for verification, to confirm whether the researchers’ interpretations accurately reflected their actual experiences. The participants raised no substantive objections to the themes; they suggested only minor wording adjustments, which were subsequently incorporated. In addition, investigator triangulation (with the first author, second author, and a methodology expert involved in coding and theme discussions) and maximum variation sampling to ensure diverse perspectives across job titles and years of distant-water fishing experience were used to cross-validate findings, enhancing the comprehensiveness and credibility of the results. Furthermore, the researchers had no personal or professional relationships with the participants, and the research team maintained a neutral stance throughout the study. Regarding transferability, this study provides detailed descriptions of the research context, participant inclusion criteria, and interview setting, enabling readers to judge the applicability of the findings to similar contexts. Regarding dependability, the research team maintained a complete audit trail, including interview records, transcripts, coding files, and theme discussion memos, which was available for external review to ensure traceability of the research process. Data saturation was continuously monitored throughout the data collection to ensure the rigor of the analysis.

### 2.7 Ethical considerations

This study was approved by the Ethics Committee of Zhoushan Hospital (Approval No. 2026-003-001) and adhered to the principles of the Declaration of Helsinki. Participants were recruited through a district-level pelagic fishery association in Zhoushan. The researchers explicitly informed all potential participants that the Association and enterprises served only as contact facilitators, had no managerial or financial involvement, and would not know which individuals participated. All participants provided written informed consent and were informed that participation was entirely voluntary and that they had the right to withdraw at any time without consequences. This information was reiterated at the beginning of each interview. Participants were informed that participation would not affect their employment, contracts, or future voyage assignments, and that they could withdraw at any time without consequences. All data were securely stored on a password-protected computer accessible only to the research team (maintained by WDD). Participant identities were anonymized using codes prior to analysis to protect privacy. To safeguard participants’ emotional safety when discussing sensitive topics such as self-management of health dilemmas, the researchers committed to pausing, rescheduling, or terminating interviews if participants experienced discomfort. Referral to hospital psychological counseling services was available if needed, although no participants required such intervention during the study. To prevent employer access to participation status or responses: (1) all interview recordings were stored on a password-protected computer accessible only to the research team; (2) transcripts were anonymized using codes before analysis; (3) the Association and enterprises did not receive any interview data, transcripts, or findings; (4) published quotations were selected to avoid combinations of details that could identify individuals in small crews. All participants provided written informed consent that explicitly included permission for the publication of anonymized quotations in academic outputs. The Ethics Committee approved the use of de-identified quotations for publication, conditional on the removal of combinations of details that could identify individuals in small crews. Transcript sharing with third parties was not covered by the original ethics approval and would require additional institutional review.

## 3 Results

All 19 participants were crew members on squid jigging vessels flying the Chinese flag and operated by two private distant-water fishing enterprises in Zhoushan City. Participants’ reported experiences were not restricted to voyages under two enterprises; given the high mobility of distant-water fishing crew in China, several participants had worked for multiple employers over their careers, and their accounts reflect cumulative experiences across different vessels and operators. The vessels shared uniform operational characteristics: target species (squid), primary fishing grounds (Southeast Pacific [*n* = 16] and Southwest Atlantic [*n* = 3]), voyage duration (approximately 20 months), crew complement (20–34 persons), absence of a qualified medical officer (first officer responsible for first aid), satellite communication availability, intermediary agency employment, and base-salary-plus-catch-share remuneration. These shared characteristics indicate that the sample represents a specific operational niche within Chinese distant-water fisheries, rather than the sector’s full diversity. To achieve the design objectives of maximum variation sampling, the sample demonstrated distributional diversity across three demographic dimensions: age (<35 years = 5, 35–49 years = 12, ≥50 years = 2), years of experience in distant-water fishing (<5 years = 11, 5–9 years = 4, ≥10 years = 4), and job rank (management-level = 5, non-management-level = 14). In subsequent thematic descriptions, participants from different age groups, years of distant-water fishing experience, and job ranks reported similar health management dilemmas within this sample, suggesting that these dilemmas may be shared among crew members in similar squid jigging operational contexts. However, given the small sample size and concentration in a single vessel type, we cannot conclude that these experiences are universal across all distant-water fishing operations. Participant demographic characteristics are presented in Table 3. Through thematic analysis, three themes and nine sub-themes were identified. The thematic structure of the self-management of health dilemmas among distant-water fishing vessel crew is presented in Table 4. Quotations are identified by unique codes (P01–P19), role category (management-level or non-management-level), and years of distant-water fishing experience (hereafter DWF).

**Table 3:** Participant demographic characteristics (*N* = 19).

Variable	Category	Value *n* (%)/median (range)
Age (years)	Median (range)	38 (26–51)
	<35	5 (26.3)
	35–49	12 (63.2)
	≥50	2 (10.5)
Marital status	Married	9 (47.4)
	Unmarried	9 (47.4)
	Divorced	1 (5.3)
Education level	Primary school	4 (21.1)
	Junior high school	8 (42.1)
	Secondary vocational school	5 (26.3)
	College	2 (10.5)
Job rank	Management-level crew	5(26.3)
	Non-management-level crew	14 (73.7)
Years of experience in distant-water fishing	Median (range)	4 (1–16)
	<5	11 (57.8)
	5–9	4(21.1)
	≥10	4 (21.1)

All participants were male distant-water fishing vessel crew members. Management-level crew included captains, second officers, and fishing masters; non-management-level crew included ordinary crew, electricians, and cooks.

**Table 4:** Thematic structure of self-management of health dilemmas among distant-water fishing vessel crew.

Themes	Sub-themes	Orem conceptual mapping
1. Inadequate distant-water medical resources [theory-consistent]	1.1 Lack of professional medical care, with crew managing health issues on their own	Therapeutic self-care demands
	1.2 Insufficient medical supplies, and self-medication difficulties	Therapeutic self-care demands
	1.3 Prolonged transport delays and delayed treatment for critical conditions	Self-care deficit
	1.4 Blocked help-seeking channels related to hierarchical pressure	Data-emergent: hierarchical pressure as structural barrier
2. High-intensity distant-water fishing operations [theory-consistent]	2.1 Disrupted work-rest cycles, high-intensity labor and chronic physical exhaustion	Self-care agency (environmental conditioning factors)
	2.2 Monotonous and isolated onboard life with emotional distress that interfered with health management	Data-emergent: emotional distress in monotonous isolated environment
	2.3 Willingness to manage health, yet constrained by onboard conditions	Self-care agency vs. self-care deficit
3. Weak interpersonal support [data-emergent]	3.1 Utilitarian peer relationships and unexpressed health concerns	Data-emergent: compromised social basic conditioning factor
	3.2 Formalized family support characterized by the pattern of “reporting good news while concealing bad news”	Data-emergent: family communication pattern

Three themes and nine sub-themes were identified, presenting the main dilemmas faced by crew of distant-water fishing vessels in health self-management.

### 3.1 Inadequate distant-water medical resources

Participants commonly reported that medical support during distant-water fishing operations was severely inadequate. Both management-level and non-management-level crew, regardless of their years of DWF experience, expressed similar dilemmas. The lack of professional medical personnel on board, insufficient medical supplies, prolonged transport times for emergency cases, and blocked help-seeking channels due to hierarchical pressure together constituted the primary barrier to crew members’ self-management of their health.

#### 3.1.1 Lack of professional medical care and crew self-management of health issues

Participants indicated that there were generally no professional healthcare providers on board. In the event of illness, they could most often rely only on their limited medical knowledge or the help of fellow crew members. Even with first-aid training, they felt helpless when facing serious illnesses.

“Even though we have received first-aid training, there is still nothing we can do when facing a serious illness on board. If this happened on land, we would definitely be able to save him.”

(P06, non-management-level crew, <5 years DWF)

“We only have medications on our boat, no healthcare personnel.”

(P10, non-management-level crew, <5 years DWF)

“There is no doctor on board, and the conditions are quite poor.”

(P09, non-management-level crew, 5–9 years DWF)

#### 3.1.2 Insufficient medical supplies and self-medication difficulties

Although the onboard medical kits were equipped with basic medications in accordance with maritime regulations, participants reported issues such as incomplete varieties and insufficient stock. More troublingly, without professional guidance, many crew members did not know which medications to take for specific symptoms.

“I have not really used the medical kit before, but I did open it once and saw some common items like Band-Aids and bandages. However, there were some medications I did not recognize.”

(P01, management-level crew, <5 years DWF)

“Once, when there was an emergency injury, we contacted a doctor remotely and were told we needed a syringe, but there was none on board.”

(P09, non-management-level crew, 5–9 years DWF)

“The medical ship does not even have the right medications. Without the right medicine, what can you do? You have to endure it.”

(P06, non-management-level crew, <5 years DWF)

#### 3.1.3 Prolonged transport delays and delayed treatment for critical conditions

When sudden, serious illnesses occurred, the limitations of the onboard environment were magnified to an extreme extent. Participants referred to ‘medical ships’—vessels equipped with medical personnel and supplies that operate in fishing grounds to provide emergency care and evacuation support for fishing crews—as their primary external medical resource. Participants described the handling of emergencies such as stroke, heart attack, and severe trauma. Transport times varied widely—when unlucky, it could take nearly a month to send a stroke patient back to China; more frustratingly, production tasks often took priority over personal health.

One participant described sending a colleague with stroke back to China:

“The fastest it takes is nearly a month.”

(P05, non-management-level crew, ≥10 years DWF).

“I felt like the boat owner delayed me. At first, I did not pay much attention because it was just a little pain. However, when it got worse, and I told him, he said we would turn back the next day.”

(P09, non-management-level crew, 5–9 years DWF)

“Even with a simple fracture, you cannot just get to a medical ship immediately. You have to finish the production work first, and then you can go.”

(P18, non-management-level crew, <5 years DWF)

“Because medical conditions on board are limited, sometimes you are far from the medical ship, and it is quite troublesome to get there.”

(P14, non-management-level crew, <5 years DWF)

“It is not very fast to send someone to a ship with a doctor. It takes a day or two, maybe two or three days, right?”

(P15, management-level crew, 5–9 years DWF)

Notably, transport times were not always lengthy. One participant reported that when a medical ship was nearby, the rescue could be quite timely: “The medical ship happened to be nearby, and it arrived in just over an hour.”

(P12, non-management-level crew, <5 years DWF)

#### 3.1.4 Blocked help-seeking channels related to hierarchical pressure

The power structure onboard placed non-management-level crew under additional psychological pressure when expressing health concerns. Participants described the authority of the captain or boat owner, and subordinates’ fear of being seen as “troublemakers,” which prevented them from promptly reporting health problems.

“I wanted to ask for help when I was sick, but the boat owner was afraid of trouble and would not let me report it.”

(P09, non-management-level crew, 5–9 years DWF)

“If you feel unwell, you first contact the second officer. Then, if the second officer assesses it as a serious problem, he contacts the captain and reports it.”

(P04, non-management-level crew, <5 years DWF)

Some participants indicated that it was possible to take leave when unwell, but the time was limited: “If you really cannot work due to illness, you can take leave, but it is usually only about two days.”

(P02, non-management-level crew, 5–9 years DWF)

### 3.2 High-intensity distant-water fishing operations

Participants generally felt that distant-water fishing operations were not just a job, but a continuous drain on both body and mind. This dilemma was evident across different job ranks and age groups. The prolonged disruption of day-night cycles, high-intensity labor, and confined environment gradually left them feeling exhausted. What made it worse was that even when they wanted to take care of themselves, the onboard conditions made it difficult to do so.

#### 3.2.1 Disrupted work-rest cycles, high-intensity labor, and chronic physical exhaustion

The work rhythm of fishing vessels was dictated by natural conditions and production demands rather than human biological rhythms. Participants described shocking working hours—during peak fishing seasons or when catches were plentiful, it was common to work continuously for more than 10 h, or even longer —and the disruption of day-night cycles was routine.

“I have been doing this for over ten years. There is no fixed schedule. When there are a lot of fish, you have to fish.”

(P08, management-level crew, ≥10 years DWF)

“Normally, the work hours are about twelve hours a day, but longer when there is more catch.”

(P17, non-management-level crew, <5 years DWF)

“It is about ten hours or so, quite long.”

(P19, non-management-level crew, <5 years DWF)

Notably, some captains proactively adjusted crew rest time in response to operational conditions. One captain stated, “As a captain, I arrange the schedule reasonably. When there are fewer fish, we call it quits early to let the crew rest.”

(P15, management-level crew, 5–9 years DWF)

#### 3.2.2 Monotonous and isolated onboard life with emotional distress that interfered with health management

The prolonged closed environment and monotonous life had a significant impact on participants’ mental health. Crew members commonly used words such as “bored,” “depressed,” and “repetitive” to describe their state. This emotional distress did not erupt suddenly but accumulated gradually over time. While participants described emotional distress as a pervasive feature of onboard life, our data do not directly establish that this distress interfered with specific health-management behaviors; rather, it represents a contextual condition that may undermine self-care agency.

“After about 13 to 15 months, I started feeling depressed. By that time, everyone had finished watching all the videos and reading all the novels they had brought. There was nothing left to pass the time.”

(P10, non-management-level crew, <5 years DWF)

“Every day is just the same thing over and over.”

(P04, non-management-level crew, <5 years DWF)

In response to this monotony, crew members developed passive coping patterns: “When I am resting, I just lie in bed watching TV.”

(P17, non-management-level crew, <5 years DWF)

The presence or absence of proactive leadership appeared to shape how crew members navigated this distress. One crew member recalled: “Sometimes I feel inexplicably irritable, but the captain talked to us and helped us accept it gradually.”

(P02, non-management-level crew, 5–9 years DWF)

This contrasted with accounts where no such support was available, leaving crew members to manage emotional challenges independently.

#### 3.2.3 Willingness to manage health, yet constrained by onboard conditions

It was noteworthy that many participants demonstrated awareness and motivation for health management: they actively took measures—buying vitamins, controlling their diet, and learning first-aid knowledge. However, in the onboard environment, these efforts often fell short.

“I bought some multivitamins and brought two or three bottles.”

(P06, non-management-level crew, <5 years DWF)

A cook who had witnessed a colleague’s heart attack expressed self-awareness: “I thought to myself, I should not end up like that. I need to take better care of my health, since it is quite troublesome out here. I need to watch my diet.”

(P16, non-management-level crew, <5 years DWF)

### 3.3 Weak interpersonal support

Participants described a deep sense of loneliness that included not only isolation from the land and separation from family but also difficulty establishing genuinely trusting relationships, even with shipmates. Both management-level and non-management-level crew members expressed this feeling of helplessness. When facing health problems, they often did not know whom to turn to for help.

#### 3.3.1 Utilitarian peer relationships and unexpressed health concerns

Although crew members lived together day in and day out, their relationships with one another often remained superficial. Participants admitted that when facing health or emotional problems, it was difficult to find someone to truly confide in. This superficial relational pattern meant that health dilemmas had to be borne in silence.

“When you are sick, it depends on your personal relationships. If you have a good relationship, you dare to speak up; if not, you have to endure it alone.”

(P06, non-management-level crew, <5 years DWF)

“It is a simple reality: you help me, I will help you. If you do not help me, I definitely will not help you, right?”

(P19, non-management-level crew, <5 years DWF)

#### 3.3.2 Formalized family support characterized by the pattern of “reporting good news while concealing bad news”

After long periods at sea, crew members’ contact with their families was reduced to a minimum. Some had not returned home for the Spring Festival for years, and others could only call home once every few months.

One crew member noted: “We have not been home for the Spring Festival for five years.”

(P07, management-level crew, 5–9 years DWF)

Even when they managed to contact their families, crew members often chose to conceal health problems. This pattern of “reporting good news while concealing bad news” effectively cut off communication with the family system that should have provided support:

“We usually do not contact family when something happens. Because when you are suffering, people say you only report good news and hide the bad, right?”

(P03, non-management-level crew, ≥10 years DWF)

“When you are away from home, you cannot just call home because you have a cold.”

(P11, management-level crew, ≥10 years DWF)

## 4 Discussion

This study revealed three types of self-management of health dilemmas among Chinese distant-water fishing vessel crew members on squid jigging operations: inadequate distant-water medical resources, high-intensity distant-water fishing operations, and weak interpersonal support. Guided by Orem’s Self-Care Theory, these correspond to unmet therapeutic self-care demands, weakened self-care agency, inadequate supportive-educative nursing systems, and resulting self-care deficits. Unlike previous quantitative studies focusing on single risk factors, this study adopted the crew members’ own perspectives to reveal the multidimensional and structural roots of these dilemmas, with attention to how deficits vary across structural positions and voyage contexts rather than assuming a uniform experience. We emphasize that our use of Orem’s Self-Care Theory is not intended to locate health deficits within individual crew members, but rather to provide a conceptual vocabulary for identifying where and how structural conditions systematically undermine individual capacity.

Beyond the regulatory gaps identified above, these practices also reflect structural power asymmetries that systematically deny crew members agency over their own health decisions. The dilemma of inadequate medical security manifested as structurally unequal access rather than simple resource absence. Non-management crew members (P04, P06, P09, P18) described passive dependency on hierarchical gatekeepers for medical decisions: “If you feel unwell, you first contact the second officer… then he contacts the captain” (P04, <5 years DWF), with production routinely prioritized over treatment—P18 (<5 years DWF) noted fractures required completing work first, while P09 (5–9 years DWF) described a boat owner who “was afraid of trouble and would not let me report it.” Management-level crew members did not describe comparable barriers to medical reporting in our data, though our sample included only five such participants and we did not systematically probe for contrasting experiences. These structural barriers align with maritime telemedicine reviews documenting that despite satellite technology enabling real-time medical consultation, most fishing vessels still lack the equipment and protocols to connect crew members with shore-based physicians, leaving onboard personnel to manage complex scenarios with minimal support (32). More striking was the geographic arbitrariness of access: P12 (<5 years DWF) reported a medical ship arriving “in just over an hour,” while P05 (≥10 years DWF) described stroke evacuation taking “nearly a month.” This geographic lottery created disparities that no individual could control. The denial of medical assistance documented by HRAS individual case investigations—refusing shore-based treatment or prioritizing catch over crew members’ health—illustrates potential patterns of concerns beyond individual self-care deficits (7). We acknowledge that these are documented cases rather than epidemiological evidence of prevalence. HRAS’s emphasis on independent satellite communication as a critical lifeline for accessing external medical support in remote fishing areas further underscores the structural nature of these dilemmas (33). Notably, P09’s experience did not immunize him against hierarchical pressure; we interpret this as suggesting that prolonged exposure may normalize such constraints, though this inference extends beyond P09’s explicit statement. This suggests accumulated experience does not translate to increased self-care capacity when basic conditioning factors remain depleted—a core source of self-care deficit per Orem’s framework. While Orem frames these as deficits produced by inadequate conditioning factors, several accounts describe practices that extend beyond resource limits to include interference with help-seeking—the prohibition of reporting (P09), the subordination of fracture treatment to production completion (P18), and reported leave restrictions (P02). These practices fall short of the minimum standards established under ILO C188, which requires ratifying States to ensure fishers’ right to timely medical treatment (Art. 29) and minimum rest periods (Art. 14). China’s non-ratification of C188 means these international standards are not legally binding domestically; however, the limited effectiveness of domestic enforcement mechanisms creates a regulatory gap where crew members have restricted formal recourse against production-prioritized medical delays or hierarchical suppression of illness reporting. While the Measures for the Administration of Fishery Crew (Art. 39) establish public reporting channels and (Art. 47) provide penalties for failure to treat sick or injured crew, participants described no awareness of these channels and no observed instance of administrative intervention. Thus, the gap is not an absence of legal duties but a disconnect between binding domestic rules, participants’ lived experience of unresponsive hierarchies, and the lack of vessel-specific inspection regimes that would activate enforcement. We flag these as raising concerns regarding alignment with international labor standards, while acknowledging that legal characterization of specific practices would require formal juridical analysis beyond the scope of this study. We note that the absence of a physician on these vessels does not by itself indicate non-compliance with ILO C188, which requires at least one crew member trained in first aid (Art. 29) rather than a qualified medical officer on every vessel. The deficit we document is not the absence of a physician per se, but the inadequacy of medical supplies, training, and communication infrastructure to enable effective first response and timely evacuation.

These accounts reveal practices that extend beyond individual self-care deficits into systemic interference with fundamental labor and human rights. The prohibition of illness reporting (P09), subordination of fracture treatment to production completion (P18), reported restriction of sick leave to 2 days (P02), and month-long delays in stroke evacuation (P05) collectively indicate serious potential gaps in the protection of crew members’ rights to medical care and safe working conditions. While Orem’s framework usefully identifies these as self-care deficits stemming from depleted basic conditioning factors, these practices also reflect structural power asymmetries that deny crew members agency over their health decisions. The vessel owner’s authority to suppress medical reporting, the prioritization of catch over treatment, and the absence of independent grievance channels are not merely resource limitations or environmental constraints; they represent systemic failures in institutional protection that leave crew members without recourse when their health needs conflict with operational demands. These findings resonate with documented cases of denied medical care and restricted communication affecting crew members and fisheries observers in the distant-water fishing sector (7), suggesting that the health dilemmas we identify are embedded within broader gaps in labor rights protections that require attention beyond individual health education or self-care capacity building.

These structural deficiencies cannot be adequately explained solely by insufficient self-care agency or individual health-management capacity. They reflect legal and managerial responsibilities distributed among multiple actors in the distant-water fishing governance chain. Fishing vessel owners and companies bear primary responsibility for ensuring adequate onboard medical resources, rest protections, and grievance channels; their failure to provide these constitutes a core source of the self-care deficits observed. Masters’ exercise of concentrated operational authority over medical reporting and evacuation decisions lacks accountability mechanisms when this authority is used to suppress crew health needs. Flag States—including China, which has not ratified C188—are responsible for establishing and enforcing domestic regulatory frameworks, including the binding duties already established under the Measures for the Administration of Fishery Crew (Art. 32–34, 35–39, 47) (18). For China, C188 serves as a comparative benchmark rather than a binding obligation. All vessels in this study flew the Chinese flag. Port States play a critical but underutilized role in inspecting the welfare conditions of fishing vessels during port calls; the absence of port State control mechanisms (Article 43) targeting medical equipment, rest records, and crew complaint procedures further erodes their protective capacity. This distributed responsibility means that the health dilemmas we document are not individual failures but rather systemic governance gaps in which multiple regulatory entry points remain unactivated.

High-intensity operations revealed a paradox of willingness structurally thwarted. Novice crew members (P04, P17, P19) described acute shock at work-rest disruption, while veterans had normalized extremes: P08 (≥10 years DWF) stated, “When there are a lot of fish, you have to fish.” Across participants, veterans appeared to have normalized extreme work rhythms; we interpret this as a possible adaptive self-care deficit in which prolonged exposure may reduce perceived pathology despite ongoing physical strain, though P08’s statement alone does not establish this mechanism. Cross-national evidence corroborates this pattern: Moroccan coastal fishermen reported a prevalence of emotional exhaustion of 16.1%, with workdays exceeding 14 h identified as the primary risk factor (34). Sleep data are equally concerning—47.2% of Moroccan fishermen met criteria for chronic insomnia, averaging only 5.5 h of sleep on workdays, with fatigue and socio-economic stress significantly predicting sleep disorder risk (35). However, proactive leadership emerged as a mitigating micro-factor: P15 deliberately adjusted schedules (“When there are fewer fish, we call it quits early”), and P02 (5–9 years DWF) recalled a captain who helped him “accept” emotional distress through conversation. These minority voices demonstrate that basic conditioning factors are not monolithic; micro-level relational practices can partially compensate for systemic deficiencies. However, such leadership was idiosyncratic rather than institutionalized—no formal mechanisms ensured rest or psychological support, underscoring that individual virtue cannot substitute for regulatory enforcement of C188 Article 14. This aligns with Sorensen et al.’s (36) finding that US fishermen averaged 3.5–4 h of sleep, and with Abaya et al.’s (37) data showing musculoskeletal problems (23.2%), gastrointestinal disorders (18.6%), and trauma (15.1%) as leading causes of repatriation. P16 (<5 years DWF), a cook who witnessed a colleague’s heart attack, exemplified the “willingness yet inability” pattern: “I need to take better care of my health… I need to watch my diet”—agency present, but structurally constrained from effective exercise.

Weak interpersonal support was not an absence but a functional decomposition of present-yet-malformed systems. Peer relationships operated through instrumental reciprocity rather than emotional trust: P19 (<5 years DWF) articulated the utilitarian logic—“you help me, I will help you”—while P06 (<5 years DWF) described conditional disclosure: “If you have a good relationship, you dare to speak up; if not, you have to endure it alone.” Even P03 (≥10 years DWF), the most experienced participant, withheld health concerns from family; tenure increased familiarity but did not generate trust in either peer or family relationships. Family support was similarly blocked by the “reporting good news while concealing bad news” pattern. A possible rank-related difference emerged, though based on limited data: P03 concealed suffering to protect the family from unalterable worry. At the same time, P11 (≥10 years DWF, management) performed resilience—“You cannot just call home because you have a cold”—suggesting hierarchical position shapes the emotional labor of family communication. Recent mixed-methods research among mobile fishers in Ghana confirms that occupational mobility generates severe relational deficits: 83.3% reported loneliness during fishing travel, manifesting as boredom (35.5%), isolation (29.1%), and anxiety (26.7%), with emotion-focused coping strategies such as alcohol consumption offering only temporary relief (38). Peer relationships and family communication operate as basic conditioning factors that shape self-care agency, rather than as components of a supportive-educative nursing system in Orem’s original formulation (26). In this study, peer networks provided instrumental aid but failed to generate the trust necessary for emotional disclosure; families provided symbolic belonging but were excluded from health information, thereby preventing crisis support. This functional decomposition—basic conditioning factors present yet compromised—represents a data-emergent pattern that illuminates how social support deficits erode self-care agency. We acknowledge that characterizing this as a novel theoretical construct beyond Orem’s original framework requires further validation. This aligns with emerging evidence: anxiety-related psychological repatriations are rising (39), and 80% of crew members in conflict zones received no company psychological support (40). At the structural level, weak interpersonal support reflects the vessel’s isolated operational environment and the absence of institutional mechanisms for crew members’ welfare. The regulatory fragmentation is acute: while IMO 2026 mental health guidelines and proposed MLC amendments explicitly exclude fishing vessels, leaving crew members in a protection vacuum where psychological distress is neither monitored nor mitigated (41).

Our findings on hierarchical pressure, blocked help-seeking, and adaptive normalization of extreme work conditions resonate with broader maritime organizational health literature. A recent mixed-methods pilot study of healthy shipping companies identified employee wellbeing, safety, work-life balance, supportive organizational culture, respectful communication, and onboard working conditions as key determinants of crew members’ health—factors largely absent in our participants’ accounts (42). While that study focused on merchant shipping rather than distant-water fishing, the contrast highlights how fishing vessel crew members operate outside the organizational health frameworks that are increasingly recognized as essential in commercial maritime sectors. This comparison underscores that the deficits we document are not merely individual self-care failures but systematic absences of supportive organizational structures that merchant shipping is beginning to institutionalize.

This two-layer framework prevents Orem’s theory from being instrumentalized to locate responsibility within individuals. By mapping deficits onto regulatory and institutional gaps—embedding contrasting cases of proactive versus absent leadership, proximate versus distant medical vessels, novice shock versus veteran normalization—we demonstrate that self-care deficits are shaped by intersecting structural and positional factors, rather than by uniform individual failure. Crew members’ purchase of vitamins, attempts at dietary control, and first-aid training confirm that agency is present; what is absent is the environment that enables its exercise. Orem recognized self-care agency is shaped by “basic conditioning factors” determined by regulatory arrangements (26); this study operationalizes that insight through integrating normative analysis with lived experience.

Several limitations should be acknowledged. The sample was recruited through a single fisheries association. It was concentrated in two enterprises operating squid-jigging vessels with uniform profiles, limiting its transferability to other vessel types, fishing grounds, or voyage durations. While several participants had worked for multiple employers over their careers, we did not systematically distinguish experiences by employer or vessel, and therefore cannot attribute specific practices to individual companies. All participants were male with predominantly junior high education or below; female crew members and higher-education perspectives are absent, and small subgroup sizes (≥50 years: *n* = 2; 5–9 years: *n* = 4) limit within-group analysis. The combination of participant codes with management status and experience categories in quotations carries a risk of deductive identification within this small, two-enterprise sample. For example, management-level crew with ≥10 years of experience are represented by only two individuals in the sample (Table 3), and certain role–category combinations (e.g., management-level, 5–9 years) are represented by a single individual. While we have replaced exact years of experience with the three categories used in Table 3 (<5 years, 5–9 years, ≥10 years) to reduce identifiability, and have selected quotations to avoid combinations of vessel-specific details (e.g., fishing ground, voyage timing, medical incident specifics) that could directly identify individuals, readers with access to enterprise crew records could potentially match demographic profiles. We have retained the (P##, role, experience category) attribution format to provide interpretive context, but acknowledge that this represents an unavoidable tension between transparency and confidentiality in small-sample qualitative research. Recruitment through the fisheries association, while necessary for access, may have introduced social desirability bias: participants may have underreported experiences of denied medical care, wage-related problems, or hierarchical pressure due to fear of indirect employer identification, despite assurances of confidentiality and employment protection. We did not formally measure self-censorship; however, the recurrence of critical accounts across participants with varying tenure and rank suggests that some degree of candid disclosure was achieved. The concentrated authority structure on board—where captains and vessel owners control employment and voyage assignments—may have amplified reluctance to disclose potentially unlawful conduct, even in private interviews away from company premises. The study is subject to recall bias, as interviews were conducted upon return or prior to departure rather than during voyages. We did not formally test for interviewer effects, though reflexive discussions were conducted after each interview. All interviews were conducted in Mandarin Chinese. Coding and initial thematic development were conducted in Chinese to preserve semantic nuance; English translation occurred only at the quotation-selection stage for manuscript preparation. While back-translation was used to check accuracy, subtle meaning loss may have occurred during translation, particularly for idiomatic expressions and emotionally laden accounts. The restricted occupational profile—all participants were squid jigging crew members from Zhoushan—limits the range of contexts captured. Finally, while maximum variation sampling achieved rank representation, hierarchical relationships may have influenced disclosure. Future research should conduct multicenter studies across major crew-exporting ports, employ longitudinal designs, adopt mixed methods to cross-validate findings, and include female crew members’ perspectives.

## 5 Conclusion

These findings, derived from squid-jigging vessel crew members in the Southeast Pacific and Southwest Atlantic fisheries, with 20-month voyages and catch-dependent remuneration, underscore that the observed self-care deficits are not attributable to individual deficiencies in health motivation or capacity. Rather, they are structurally produced through the systematic deprivation of basic conditioning factors identified in Orem’s theory—inadequate health care systems, hazardous occupational environments, and insufficient resource availability—and shaped by intersecting structural and positional factors including rank, voyage context, and geographic arbitrariness. Notably, interpersonal support was not absent but functionally decomposed: peer and family systems were present yet unable to integrate into effective care. We interpret this as a data-emergent pattern in which compromised basic conditioning factors (social support) erode self-care agency, rather than as a distinct type of self-care deficit in Orem’s original theoretical terms. We caution against generalizing these findings to all Chinese distant-water fishing operations, given the concentration of our sample in this specific operational niche.

Recommendations directly grounded in participants’ stated experiences and priorities include: (1) independent and confidential medical reporting channels that bypass captains and owners, addressing the hierarchical blockage described by P04, P09, and P18; (2) enforced work-rest protections with accountability mechanisms, responding to the routine disruption of biological rhythms documented across all experience levels; (3) accessible medication guidance for non-medical crew members, addressing uncertainty about medication use reported by multiple participants (P01, P06); (4) competent onboard first-aid capacity, responding to participants’ expressed helplessness when serious illness struck despite existing training (P06); and (5) reliable telemedical advice via existing satellite communication infrastructure, building on remote consultation attempts that failed due to missing supplies or guidance (P09).

Broader system-level recommendations, supported by regulatory analysis and external maritime health literature but requiring feasibility assessment beyond this dataset, include: ratification and implementation of ILO C188, supplemented by additional reforms addressing mental health protection and harassment prevention, since these are not explicit standalone provisions in the Convention; enhancement of fishing-vessel-specific inspection and enforcement mechanisms; improved activation of enforcement and compliance mechanisms for existing domestic regulatory frameworks—including the Measures for the Administration of Fishery Crew (Art. 32–34, 35–39, 47)—to ensure that binding protections already established on paper are realized in practice, while using C188 as a non-binding benchmark to identify gaps requiring supplementary reform; and exploration of accountability mechanisms for crew members’ medical assistance. These structural changes align with the organizational health determinants identified in merchant shipping research (42), while recognizing the distinct occupational structure of distant-water fishing vessels.

Future research should conduct multicenter comparative, intervention, and longitudinal studies and include female crew members’ perspectives to enhance the transferability of the findings.

## Data Availability

The datasets presented in this article are not readily available because raw audio recordings and full transcripts cannot be shared due to the sensitive nature of the accounts and the risk of deductive identification in this small, concentrated sample. De-identified thematic codes and selected anonymized quotations may be provided to qualified researchers upon request to the corresponding author, pending verification of research purpose, ethical approval, and compliance with institutional data protection protocols. Requests to access the datasets should be directed to Boer Yan, zsyhb@163.com.
