# Supplementary material for: Self-management of health dilemmas among Chinese distant-water fishing vessel crew: a descriptive qualitative study based on Orem’s self-care theory
Source: Front Public Health. 2026 Sep 22;14:1928192. doi: 10.3389/fpubh.2026.1928192 (PMC13638627; doi:10.3389/fpubh.2026.1928192)
Supplement: Supplementary file 1 [file Supplementary_file_1.DOCX]

Supplementary Material S1:

**Semi-Structured Interview Guide**

1. Opening Statement

Hello, we are researchers from Zhoushan Hospital. Thank you for taking the time to participate in this interview.

We are conducting a study on the self-management of health dilemmas of the crew of distant-water fishing vessels. The purpose is to understand how you take care of your physical and mental health during ocean-going operations, and what practical difficulties you encounter when managing your health onboard. Your honest sharing is very important to us.

This interview will take approximately 30 to 60 minutes. The interview content will be used solely for academic research. All your responses will be kept strictly confidential and will not be disclosed to your company, colleagues, or any third party. In the final report, your identity information will be anonymized, and no content that could identify you personally will appear.

Your participation is entirely voluntary. You have the right to pause or terminate the interview at any time, without providing any reason, and without any consequences. Do you agree to participate and to have the interview audio-recorded?

2. Demographic Information Collection Form

Age: ___________ Gender: ___________ Marital Status: ___________

Education Level: ___________ Job Title: ___________

Years of Experience in Distant-Water Fishing: ___________

3. Core Questions

(1) Perceived Health Threats and Self-Care Needs

1. What health problems do you worry about most during distant-water operations?

Probing prompt: What health problems have you experienced yourself? Have you seen or heard of any health problems among your colleagues?

2. In what situations do you think you are most likely to get injured or become ill onboard?

Probing prompt: Could you tell me more about what happened in that situation?

3. What do you think you need to do to maintain your health while onboard?

Probing prompt: What differences do you see between staying healthy onboard and staying healthy on land?

(2) Environmental and Occupational Factors Constraining Self-Care Agency

4. How is your work-rest schedule arranged onboard? How does this rhythm affect your physical health or mood?

Probing prompt: Do you find the work intensity high? Can you get enough sleep and rest?

5. What do you know about the medical kit or first-aid equipment onboard? Do you use them in daily practice?

Probing prompt: Do you know what medications are inside? Do you know which medications are for which conditions? Do you bring any common medications yourself?

6. If you or a colleague feels unwell on board, how do you usually handle it? Who do you turn to for help?

Probing prompt: Who would you think of first? Was the help-seeking process smooth? Did you encounter any difficulties?

(3) Experiences of Self-Care Deficit and Help-Seeking Difficulties

7. Have you encountered or heard of any situations onboard where illness or injury was particularly difficult to manage? What happened?

Probing prompt: How was it handled? What was the outcome?

8. When you feel emotionally distressed onboard, how do you usually cope? Is there anyone you can talk to?

Probing prompt: whom would you talk to? Or do you keep it to yourself? Why?

9. In your opinion, what aspects of the current onboard conditions are insufficient for protecting your health?

Probing prompt: What do you think companies, hospitals, or the government could do to improve things? Do you have any suggestions?

4. Closing Statement

That covers the main content of today’s interview. Thank you for taking the time to share with us. Your insights are very helpful in understanding the real health conditions and needs of crew members.

If we have any further questions later, may we contact you again?

If you are interested in the findings of this study, you are welcome to contact us after the study is completed.

Wishing you good health and safe voyages! Thank you!
