# Supplementary material for: Self-management of health dilemmas among Chinese distant-water fishing vessel crew: a descriptive qualitative study based on Orem’s self-care theory
Source: Front Public Health. 2026 Sep 22;14:1928192. doi: 10.3389/fpubh.2026.1928192 (PMC13638627; doi:10.3389/fpubh.2026.1928192)
Supplement: Supplementary file 2 [file Supplementary_file_2.DOCX]

Supplementary Material S2:

## **Title:** Self-Management of Health Dilemmas Among Chinese Distant-Water Fishing Vessel Crew: A Descriptive Qualitative Study Based on Orem’s Self-Care Theory

**COREQ Checklist**

| **No** | **Item** | **Guide Questions/Description** | **Reported in Section** |
| --- | --- | --- | --- |
| **Domain 1: Research Team and Reflexivity** | | | |
| Personal Characteristics | | | |
| 1 | Interviewer/facilitator | Which author/s conducted the interview or focus group? | 2.4 Data collection |
| 2 | Credentials | What were the researcher’s credentials? | 2.4 Data collection |
| 3 | Occupation | What was their occupation at the time of the study? | 2.4 Data collection |
| 4 | Gender | Was the researcher male or female? | 2.4 Data collection |
| 5 | Experience and training | What experience or training did the researcher have? | 2.4 Data collection |
| Relationship with Participants | | | |
| 6 | Relationship established | Was a relationship established prior to study commencement? | 2.4 Data collection |
| 7 | Participant's knowledge of the interviewer | What did the participants know about the researcher? | 2.4 Data collection |
| 8 | Interviewer characteristics | What characteristics were reported about the interviewer/facilitator? | 2.4 Data collection |
| **Domain 2: Study Design** | | | |
| Theoretical Framework | | | |
| 9 | Methodological orientation and theory | What methodological orientation was stated to underpin the study? | 2.1 Study design; 2.3 Interview guide development |
| Participant Selection | | | |
| 10 | Sampling | How were participants selected? | 2.2 Participants and setting |
| 11 | Method of approach | How were participants approached? | 2.2 Participants and setting |
| 12 | Sample size | How many participants were in the study? | 2.2 Participants and setting;3 Results |
| 13 | Non-participation | How many people refused to participate or dropped out? Reasons? | 2.2 Participants and setting |
| Setting | | | |
| 14 | Setting of data collection | Where were the data collected? | 2.3 Interview guide development; 2.4 Data collection |
| 15 | Presence of non-participants | Was anyone else present besides the participants and researchers? | 2.4 Data collection |
| 16 | Description of sample | What are the important characteristics of the sample? | Table 2; 3 Results |
| Data Collection | | | |
| 17 | Interview guide | Did the authors provide questions, prompts, or guides? Was it pilot tested? | 2.3 Interview guide development; Table 1 |
| 18 | Repeat interviews | Were repeat interviews carried out? If yes, how many? | Not conducted |
| 19 | Audio/visual recording | Did the research use audio or visual recording to collect the data? | 2.4 Data collection |
| 20 | Field notes | Were field notes made during and/or after the interview or focus group? | 2.4 Data collection |
| 21 | Duration | What was the duration of the interviews or focus group? | 2.4 Data collection |
| 22 | Data saturation | Was data saturation discussed? | 2.2 Participants and setting |
| 23 | Transcripts returned | Were transcripts returned to participants for comment and/or correction? | 2.5 Data analysis |
| **Domain 3: Analysis and Findings** | | | |
| Data Analysis | | | |
| 24 | Number of data coders | How many data coders coded the data? | 2.5 Data analysis |
| 25 | Description of the coding tree | Did the authors describe the coding tree? | Supplementary Material S3 |
| 26 | Derivation of themes | Were themes identified in advance or derived from the data? | 2.5 Data analysis |
| 27 | Software | What software, if applicable, was used to manage the data? | 2.5 Data analysis |
| 28 | Participant checking | Did participants provide feedback on the findings? | 2.6 Trustworthiness and rigor |
| Reporting | | | |
| 29 | Quotations presented | Were participant quotations presented to illustrate the themes/findings? Was each quotation identified? | 3.1–3.3 Results |
| 30 | Data and findings are consistent | Was there consistency between the data presented and the findings? | 2.5 Data analysis; 2.6 Trustworthiness and rigor |
| 31 | Clarity of major themes | Were major themes clearly presented in the findings? | Table 3: 3.1–3.3 Results |
| 32 | Clarity of minor themes | Is there a description of diverse cases or a discussion of minor themes? | 3.1–3.3 Results |

Reference: Tong A, Sainsbury P, Craig J. Consolidated criteria for reporting qualitative research (COREQ): a 32-item checklist for interviews and focus groups. Int J

Qual Health Care. (2007) 19:349–57. doi: 10.1093/intqhc/mzm042.
