# Supplementary material for: Self-management of health dilemmas among Chinese distant-water fishing vessel crew: a descriptive qualitative study based on Orem’s self-care theory
Source: Front Public Health. 2026 Sep 22;14:1928192. doi: 10.3389/fpubh.2026.1928192 (PMC13638627; doi:10.3389/fpubh.2026.1928192)
Supplement: Supplementary file 3 [file Supplementary_file_3.DOCX]

Supplementary Material S3:

## Title: Self-Management of Health Dilemmas Among Chinese Distant-Water Fishing Vessel Crew: A Descriptive Qualitative Study Based on Orem’s Self-Care Theory

**Analytical approach:** Thematic analysis (Braun and Clarke, 2006)

**Software:** NVivo 15.0

**Coding Tree Structure**

| **Themes** | **Sub-themes** | **Representative Quotes (Participant Sources)** | **Orem Concept** | **Label** |
| --- | --- | --- | --- | --- |
| Theme 1: Inadequate distant-water medical resources | 1.1 Lack of professional medical care and crew self-management of health issues | "Even though we have received first-aid training, there is still nothing we can do when facing a serious illness on board. If this happened on land, we would definitely be able to save him." (P06, non-management-level crew, <5 years DWF)  “We only have medications on our boat, no healthcare personnel.” (P10, non-management-level crew, <5 years DWF)  "There is no doctor on board, and the conditions are quite poor." (P09, non-management-level crew, 5–9 years DWF) | Therapeutic Self-Care Demands | Theory-consistent |
|  | 1.2 Insufficient medical supplies, and self-medication is difficult | “I have not really used the medical kit before, but I did open it once and saw some common items like Band-Aids and bandages. However, there were some medications I did not recognize." (P01, management-level crew, <5 years DWF)  “Once, when there was an emergency injury, we contacted a doctor remotely and were told we needed a syringe, but there was none on board.” (P09, non-management-level crew, 5–9 years DWF)  "The medical ship does not even have the right medications. Without the right medicine, what can you do? You have to endure it." (P06, non-management-level crew, <5 years DWF) | Therapeutic Self-Care Demands | Theory-consistent |
|  | 1.3 Prolonged transport delays and delayed treatment for critical conditions | One participant described sending a colleague with stroke back to China: "The fastest it takes is nearly a month.” (P05, non-management-level crew, ≥10 years DWF).  "I felt like the boat owner delayed me. At first, I did not pay much attention because it was just a little pain. But when it got worse, and I told him, he said we would turn back the next day." (P09, non-management-level crew, 5–9 years DWF)  "Even with a simple fracture, you cannot just get to a medical ship immediately. You have to finish the production work first, and then you can go." (P18, non-management-level crew, <5 years DWF)  "Because medical conditions on board are limited, sometimes you are far from the medical ship, and it is quite troublesome to get there." (P14, non-management-level crew, <5 years DWF)  "It is not very fast to send someone to a ship with a doctor. It takes a day or two, maybe two or three days, right?" (P15, management-level crew, 5–9 years DWF)  Notably, transport times were not always lengthy. One participant reported that when a medical ship was nearby, the rescue could be quite timely: “The medical ship happened to be nearby, and it arrived in just over an hour.” (P12, non-management-level crew, <5 years DWF) | Self-Care Deficit | Data-emergent |
|  | 1.4 Blocked help-seeking channels related to hierarchical pressure | "I wanted to ask for help when I was sick, but the boat owner was afraid of trouble and would not let me report it." (P09, non-management-level crew, 5–9 years DWF)  “If you feel unwell, you first contact the second officer. Then, if the second officer assesses it as a serious problem, he contacts the captain and reports it.” (P04, non-management-level crew, <5 years DWF)  Some participants indicated that it was possible to take leave when unwell, but the time was limited: "If you really cannot work due to illness, you can take leave, but it is usually only about two days." (P02, non-management-level crew, 5–9 years DWF) | Data-emergent: Hierarchical pressure as structural barrier | Data-emergent |
| Theme 2: High-intensity distant-water fishing operations | 2.1 Disrupted work-rest cycles, high-intensity labor, and chronic physical exhaustion | "I have been doing this for over ten years. There is no fixed schedule. When there are a lot of fish, you have to fish." (P08, management-level crew, ≥10 years DWF)  "Normally, the work hours are about twelve hours a day, but longer when there is more catch." (P17, non-management-level crew, <5 years DWF)  "It is about ten hours or so, quite long." (P19, non-management-level crew, <5 years DWF)  Notably, some captains proactively adjusted crew rest time in response to operational conditions. One captain stated, "As a captain, I arrange the schedule reasonably. When there are fewer fish, we call it quits early to let the crew rest." (P15, management-level crew, 5–9 years DWF) | Self-Care Agency (Environmental) | Theory-consistent |
|  | 2.2 Monotonous and isolated onboard life with emotional distress that interfered with health management | “After about 13 to 15 months, I started feeling depressed. By that time, everyone had finished watching all the videos and reading all the novels they had brought. There was nothing left to pass the time.” (P10, non-management-level crew, <5 years DWF)  “Every day is just the same thing over and over.” (P04, non-management-level crew, <5 years DWF)  In response to this monotony, crew members developed passive coping patterns: "When I am resting, I just lie in bed watching TV." (P17, non-management-level crew, <5 years DWF)  The presence or absence of proactive leadership appeared to shape how crew members navigated this distress. One crew member recalled: "Sometimes I feel inexplicably irritable, but the captain talked to us and helped us accept it gradually." (P02, non-management-level crew, 5–9 years DWF) | Data-emergent: Emotional distress in monotonous isolated environment | Data-emergent |
|  | 2.3 Willingness to manage health, yet constrained by onboard conditions | “I bought some multivitamins and brought two or three bottles.” (P06, non-management-level crew, <5 years DWF)  A cook who had witnessed a colleague's heart attack expressed self-awareness: "I thought to myself, I should not end up like that. I need to take better care of my health, since it is quite troublesome out here. I need to watch my diet." (P16, non-management-level crew, <5 years DWF) | Self-Care Agency vs. Deficit | Theory-consistent |
| Theme 3: Weak interpersonal support | 3.1 Utilitarian peer relationships and unexpressed health concerns | "When you are sick, it depends on your personal relationships. If you have a good relationship, you dare to speak up; if not, you have to endure it alone." (P06, non-management-level crew, <5 years DWF)  "It is a simple reality: you help me, I will help you. If you do not help me, I definitely will not help you, right?" (P19, non-management-level crew, <5 years DWF) | Data-emergent: Compromised social basic conditioning factor | Data-emergent |
|  | 3.2 Formalized family support characterized by the pattern of “reporting good news while concealing bad news” | One crew member noted: "We have not been home for the Spring Festival for five years." (P07, management-level crew, 5–9 years DWF)  Even when they managed to contact their families, crew members often chose to conceal health problems. This pattern of "reporting good news while concealing bad news" effectively cut off communication with the family system that should have provided support: "We usually do not contact family when something happens. Because when you are suffering, people say you only report good news and hide the bad, right?" (P03, non-management-level crew, ≥10 years DWF)  "When you are away from home, you cannot just call home because you have a cold." (P11, management-level crew, ≥10 years DWF) | Data-emergent: Family communication pattern | Data-emergent |

**Coding Process Overview**

| **Phase** | **Description** |
| --- | --- |
| **Phase 1: Familiarization with the data** | The first author (WDD) repeatedly read all 19 interview transcripts and reviewed field notes and research memos. |
| **Phase 2: Generating initial codes** | The first author systematically coded all 19 transcripts using an initial coding framework developed based on the core concepts of Orem’s Self-Care Theory (self-care agency, self-care demands, self-care deficit). To ensure coding rigor, the second author independently coded five of the transcripts (approximately 25%). Coding concordance and discrepancies were calibrated and resolved through discussions within the research team, including a methodology expert. The final coding framework was then uniformly applied to the entire dataset by the first author. NVivo 15.0 was used for data management. |
| **Phase 3: Searching for themes** | Based on the coding framework, codes with similar meanings were grouped to form nine sub-themes. |
| **Phase 4: Reviewing themes** | By comparing the interrelationships among the sub-themes, they were further synthesized into three themes. |
| **Phase 5: Defining and naming themes** | Themes were refined and named through team consensus. |
| **Phase 6: Producing the report** | Final themes and sub-themes were determined and presented with representative quotations. |

**Reference:** Braun V, Clarke V. Using thematic analysis in psychology. Qual Res Psychol. (2006) 3:77–101. doi: 10.1191/1478088706qp063oa.
